# Trends in severe functional limitations among working and non-working adults in Germany: Towards an (un)-healthy working life?

**DOI:** 10.1007/s10433-024-00809-x

**Published:** 2024-04-23

**Authors:** Johannes Beller, Stefanie Sperlich, Jelena Epping, Juliane Tetzlaff

**Affiliations:** Medical Sociology Unit, Center for Public Health and Health Care, Hannover Medical School, Carl-Neuberg-Str. 1, 30625 Hannover, Germany

**Keywords:** Disability, Functional limitations, Morbidity, Trends, Work, Healthy working life expectancy

## Abstract

We examined trends in severe functional limitations among working and non-working adults in Germany (ages 40–65). Four population-based samples of 11,615 participants were used, spanning the time periods 2002–2021. The overall prevalence of severe limitations was found to be 12.8% in the sample, but also varied from 10 to 20% according to occupational group. Over time, severe limitations were found to have increased, from 10.6% in 2002 to 13.2% in 2021. Logistic regression analysis showed that severe limitations increased significantly in certain subgroups, including working women with a low skilled white collar occupational group, working men with a low skilled blue collar occupational group and, particularly, among the whole non-working population, whereas limitations remained largely the same in the other groups, including most of the working population. In terms of expectancies, overall working life expectancy increased. Along with this increase, healthy (non-severely limited) working life expectancy increased, but this trend was accompanied by a clear increase in unhealthy working life expectancy (severely limited). Thus, although severe limitations have increased in some groups in the working-age adults, people today can expect to work more years free from severe limitations than before. In the future, potentials to increase working life expectancy may come to an end, as severe limitations increased strongly in the non-working population, which could limit the prospects for a further increase in the proportion of the population in employment. Further studies are needed to investigate the potential impact of the increasing prevalence of severe limitations on the population’s ability to work.

Functional limitations are difficulties an individual might have in performing vital everyday activities. This could include limitations in performing single or multiple activities of daily living like dressing, walking or shopping. Additionally, limitations among the middle-aged are often related to difficulties in performing work, often resulting in absenteeism or early retirement (Cabrero-García et al. 2020, 2021; Jagger et al. 2010). Limitations can be classified into non-severe and severe based on the degree of difficulty experienced by the individual (Verbrugge and Jette 1994). Non-severe limitations are those that cause some difficulty but generally do not prevent the individual from performing the activity independently or with minimal assistance. Severe limitations, on the other hand, are those in which the individual experiences severe difficulties such that generally substantial assistance is required, or the individual cannot perform the activity at all (Meulenkamp et al. 2019). Limitations can have serious consequences for the health and well-being of individuals. In large longitudinal studies limitations strongly predict increased psychological distress, institutionalization, and mortality (Kurichi et al. 2017; Mezuk et al. 2012; St John et al. 2014). Severe limitations can also reduce the work ability and working life of the affected individual, by limiting the range of tasks that the individual can perform and by increasing the need for support at work and thus may also prohibit the individual from working at all (Bugajska et al. 2020; Volberding et al. 2019). Many studies have investigated trends in overall limitations; however, despite the importance of severe limitations for population health and the feasibility of extending working lives, there is limited evidence on severe limitation trends and their consequences in limiting working life among working-age adults (Parker et al. 2020).

Existing studies on trends in limitations have focused mostly on older adults and analysed overall limitations (Crimmins 2004; Jagger et al. 2016; Lee et al. 2020; Robine andJagger 2017). However, fewer studies have analyzed trends in working-age adults, despite many being affected by activity limitations (Freedman et al. 2013; Martin et al. 2010; Rubio Valverde et al. 2022; van der Noordt et al. 2023) or have focused on trends in severe limitations. Unlike studies in older adults, studies in working-aged adults (i.e., adults aged < 65 years) have recently found evidence of increasing limitations over time. For example, in the US, Zajacova and colleagues (2018) found that functional limitations' prevalence has increased over time, partly due to countervailing forces such as rising psychological distress, income difficulties, obesity, and alcohol use, which were countered by increasing educational attainment and healthy behaviours. In Europe, Beller and colleagues (2022a, b; 2020) found that limitations increased over time in those aged < 65 years but did not consider differences according to working status or working group. Recently, van der Noordt et al. (2019), analysed trends in working life expectancy with and without disability in workers aged 55–65 in the Netherlands from 1992 to 2016. Using the Global Activity Limitations Indicator (GALI) to measure overall disability, they found that overall disability increased over time in workers and that workers with disability worked longer over time, no matter their gender or education level. However, more research is needed to specifically analyse trends in severe limitations among working-age adults, especially because severe limitations have the potential to severely limit individuals’ working ability.

From a theoretical perspective two theoretical frameworks might help understand trends and consequences of severe functional limitations among working-age adults: the healthy worker effect and role theory. The healthy worker effect refers to the phenomenon that workers tend to be healthier than non-workers due to a selection process that excludes unhealthy individuals from working (Li and Sung 1999). Therefore, it is to be expected that non-working adults may have higher levels of severe functional limitations because they are more likely to be limited enough to have stopped working or to never have worked. Additionally, the healthy worker effect may also predict that potential increasing trends in severe functional limitations reported by previous studies might be focused among non-working adults over time, as more individuals without or with non-severe functional limitations may continue working due to economic incentives or social policies that encourage longer working lives (Ní Léime et al. 2020). Furthermore, role theory might help interpret consequences of reduced work participation in those with functional limitations. Role theory suggests that work is an important source of identity, meaning, and social integration for individuals, and that losing or lacking work can have negative consequences for their health and well-being (Barnett and Gareis 2006; van der Noordt et al. 2014). Therefore, working-age adults with severe functional limitations may experience additional distress if they are not working. In addition to the barriers already present due to having functional limitations (Bonaccio et al. 2020), this distress may further affect their health and well-being, emphasizing the need to investigate how work and functional health trends have evolved over time.

To our knowledge, no previous study has examined trends of severe limitations among working and non-working adults in Germany, a gap that we aim to fill with this study. It goes beyond most previous studies on trends in limitations by explicitly considering trends in severe limitations among working-age adults and by further investigating how these trends differ according to employment status and occupational group. Four cross-sectional population-based samples (*N* = 11,615) of German Adults are used spanning the time periods 2002–2021.

## Methods

### Sample

Data were drawn from public releases of the German Aging Survey (Klaus et al. 2017; Vogel et al. 2023). The German Aging Survey (Deutscher Alterssurvey; DEAS) is a cohort-sequential longitudinal, population-based study on Germans aged 40 years and older that is provided by the Research Data Center of the German Center of Gerontology (Klaus et al. 2017). For the DEAS, baseline first-time participants are drawn randomly by probability sampling in 1996, 2002, 2008 and 2014. Additionally, participants from previous waves were re-contacted if possible. The newest 2021 wave consisted only of repeat respondents. All interviews are conducted face-to-face in the participant’s residence, with the exception of the 2021 wave, which included a telephone-based interview due to the COVID-19 pandemic. All procedures are in accordance with German law and the ethical standards of the 1964 Helsinki declaration and its later amendments. We used the data of participants who filled out a drop-off questionnaire and were 40–65 years old; furthermore, we included only the first-time participants in 2002, 2008, and 2014, and additionally included the 2021 wave, which consisted of repeat respondents. The 2002 wave was the first one to include the measure of physical functioning. Thus, in our analysis, the cross-sectional independent baseline samples of 2002, 2008, and 2014 were used; additionally, we used the 2021 wave, which consisted only of repeat respondents. After excluding participants with missing values listwise (about 1% of the sample), a final sample with N = 11,615 participants resulted (N_2002_ = 2335; N_2008_ = 3564; N_2014_ = 3512; N_2021_ = 2204). Ethics board approval was not required, because we only conducted analyses of completely anonymized DEAS-datasets. Written informed consent was obtained from all subjects before the study.

### Measures

To measure severe limitations the thoroughly validated and widely used Physical Functioning subscale of the Short Form 36 Health Survey was used (Bohannon and DePasquale 2010; Bullinger 1995; Hays et al. 1993). It assesses the degree of limitation due to health problems in a range of everyday activities such on a 3-point scale ranging from “severely limited” to “somewhat limited” to “not limited at all”. Individuals were classified as severely limited if they reported to be severely limited in at least one of the following activities: “Moderate activities, such as moving a table, pushing a vacuum cleaner, bowling, or playing golf”, “Lifting or carrying groceries”, “Climbing several flights of stairs”, “Climbing one flight of stairs”, “Bending, kneeling, or stooping”, “Walking more than one kilometer”, “Walking several blocks”, “Walking one block”, “Bathing or dressing yourself”. Additionally, age, gender, working status (0 = currently not employed or self-employed; 1 = currently employed or self-employed) and occupational group were included in the analyses. Regarding occupational groups, we distinguished between high skilled white collar (WC-HS), low skilled white collar (WC-LS), high skilled blue collar (BC-HS), and low skilled blue collar (BC-LS) occupational groups based on the ISCO 1-level coding of the current or, alternatively, last job as reported by the participant (High skilled white collar: ISCO 1–3; Low skilled white collar: ISCO 4–5; High skilled blue collar: ISCO 6–7; Low skilled blue collar: ISCO 8–9; Missing: No information regarding last occupational group available). The occupational group for (currently) non-working participants is based on information of their last job, as reported by the participants.

### Data analysis

First, descriptive statistics of all variables are reported. Then, to determine trends across time, multiple logistic regression analyses are conducted predicting limitations as the outcome by time period for the whole sample controlled for age and stratified by gender. Then, further stratified logistic regression analyses were conducted to depict time trends in severe limitations according to working status and occupational groups, controlling for age using the weights provided by the German Aging Survey. In the regression analyses, time period was treated as a continuous variable and scaled in such a way that 0 = 2002 and 1 = 2021. Additionally, in accordance with previous studies, trends in life years individuals can expect to be in paid work (Working Life Expectancy; WLE) and trends in life years individuals can expect to be healthy, which in this case is defined as being free of severe limitations and in paid work were calculated (Healthy Working Life Expectancy; HWLE) based on the observed age-specific proportions of working / non-working and disabled / non-disabled populations of age 40 to 65 employing the widely-used Sullivan method (Imai and Soneji 2007). Concretely, to calculate the expectancies we determined the proportion of participants working with and without severe limitations at each age and time-period. Next, we calculated WLE by summing the proportions of the work participations across age groups. This yielded the expected number of remaining years of work. Similarly, we calculated HWLE by summing the proportions of those working without severe limitations across age groups. In line with previous studies, mortality was ignored, which means that WLE expresses the average number of years spent in the labour market for those surviving up to age 65 (Boissonneault and Rios 2021; Lievre et al. 2007). HWLE as a subset of WLE represents the number of life years to be expected to be free of severe limitations and in paid work. Accordingly, the difference between WLE and HWLE represents the number of years an individual can be expected to be in paid work while reporting a severe limitation (Unhealthy Working Life Expectancy, UHWLE). Due to small sample size in some subgroups (e.g., women working in BC-HS occupations) the data from the two earlier time points (2002 and 2008) and the data from the two later time points (2014 and 2021) are combined by treating the observations from both time points as independent for the expectancy calculations. As such, for the HWLE analyses the two periods 2002–2008 and 2014–2021 are compared. All analyses were conducted with R.

## Results

Overall, as seen in Table 1, participants were on average 54.05 years old (SD = 7.19), with 52.2% being female and 67.4% being in active work. About 12.8% of the sample reported to have a severe limitation. Prevalence of limitations, however, varied widely according to occupational group from 10% (WC-HS) to 20% (BC-LS). Furthermore, as seen in Table 2, age-unadjusted prevalence of limitations descriptively increased over time from 10.6% in 2002 to 13.2% in 2021 (descriptive differences according to working status are displayed in appendix Table 3).

**Table 1:** Severe limitations and socio-demographics in german working age adults across occupational groups

	Stratified by occupational group					
	Overall	WC-HS	WC-LS	BC-HS	BC-LS	Missing
N	11,615	5666	2433	1553	1517	446
Severe limitations (%)	12.8	10.0	14.8	13.2	20.0	11.0
Age (mean (SD))	54.05 (7.19)	54.29 (7.16)	54.08 (7.14)	53.74 (7.29)	53.58 (7.18)	53.62 (7.46)
Gender = Women (%)	52.2	53.2	74.4	16.7	47.8	57.4
Working status = Working (%)	67.4	74.4	62.7	62.2	60.0	47.5
Education (%)						
Lower	7.2	1.3	6.3	6.2	27.8	20.2
Intermeditate	52.8	34.0	76.2	74.6	66.0	42.8
Higher	40.1	64.8	17.6	19.1	6.3	37.0
Occupational group (%)						
WC-HS	48.8	100.0	0.0	0.0	0.0	0.0
WC-LS	20.9	0.0	100.0	0.0	0.0	0.0
BC-HS	13.4	0.0	0.0	100.0	0.0	0.0
BC-LS	13.1	0.0	0.0	0.0	100.0	0.0
Missing	3.8	0.0	0.0	0.0	0.0	100.0

WC-HS, High skilled white collar workers; WC-LS, Low skilled white collar workers; BC-HS, High skilled blue collar workers; BC-LS, Low skilled blue collar workers; Missing, Missing occupational information

**Table 2:** Severe limitations and socio-demographics in german working-age adults across time periods (2002–2021)

	Stratified by time period			
	2002	2008	2014	2021
N	2335	3564	3512	2204
Severe limitations (%)	10.6	9.8	17.0	13.2
Age (mean (SD))	52.34 (7.63)	52.67 (7.17)	53.91 (7.04)	58.32 (4.93)
Gender = Women (%)	49.4	52.8	52.0	54.7
Working status = Working (%)	59.9	67.1	70.2	71.2
Education (%)				
Lower	14.6	6.9	5.3	2.7
Intermeditate	53.3	54.7	53.2	48.5
Higher	32.1	38.4	41.5	48.9
Occupational group (%)				
WC-HS	37.5	47.1	50.8	60.3
WC-LS	22.5	20.5	21.0	19.9
BC-HS	18.2	14.0	12.2	9.1
BC-LS	16.9	14.2	12.4	8.3
Missing	4.9	4.2	3.6	2.5

WC-HS, High skilled white collar workers; WC-LS, Low skilled white collar workers; BC-HS, High skilled blue collar workers; BC-LS, Low skilled blue collar workers; Missing, Missing occupational information

**Fig. 1:**
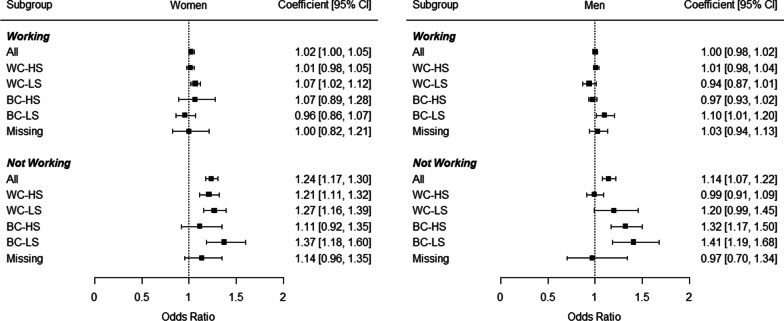
Trend coefficients for severe limitations in Germany for working-age adults across subgroups. *Notes*: Analyses were adjusted for age. WC-HS, High skilled white collar workers; WC-LS, Low skilled white collar workers; BC-HS, High skilled blue collar workers; BC-LS, Low skilled blue collar workers; Missing, Missing occupational information

Next, weighted multiple logistic regression analyses were conducted to study time trends for severe limitations adjusted for age. While severe limitations did not change significantly in most working groups, severe limitations significantly increased in working women with an WC-LS occupational group as well as currently non-working women in general (Fig. 1). In men, limitations significantly increased in working men with a BC-LS occupational group as well as currently non-working men in general.

**Fig. 2:**
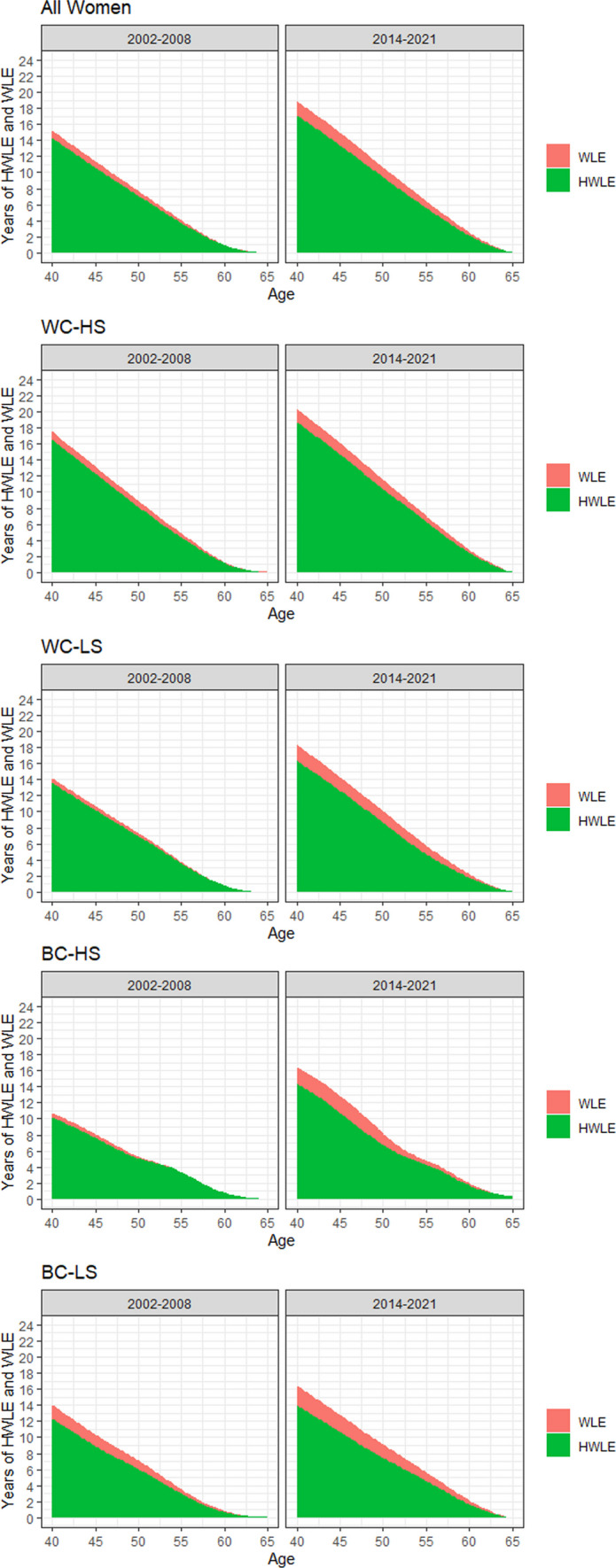
Changes in WLE and HWLE in terms of severe limitations at age 40 and over in women. *Notes*: WC-HS, High skilled white collar workers; WC-LS, Low skilled white collar workers; BC-HS, High skilled blue collar workers; BC-LS, Low skilled blue collar workers

**Fig. 3:**
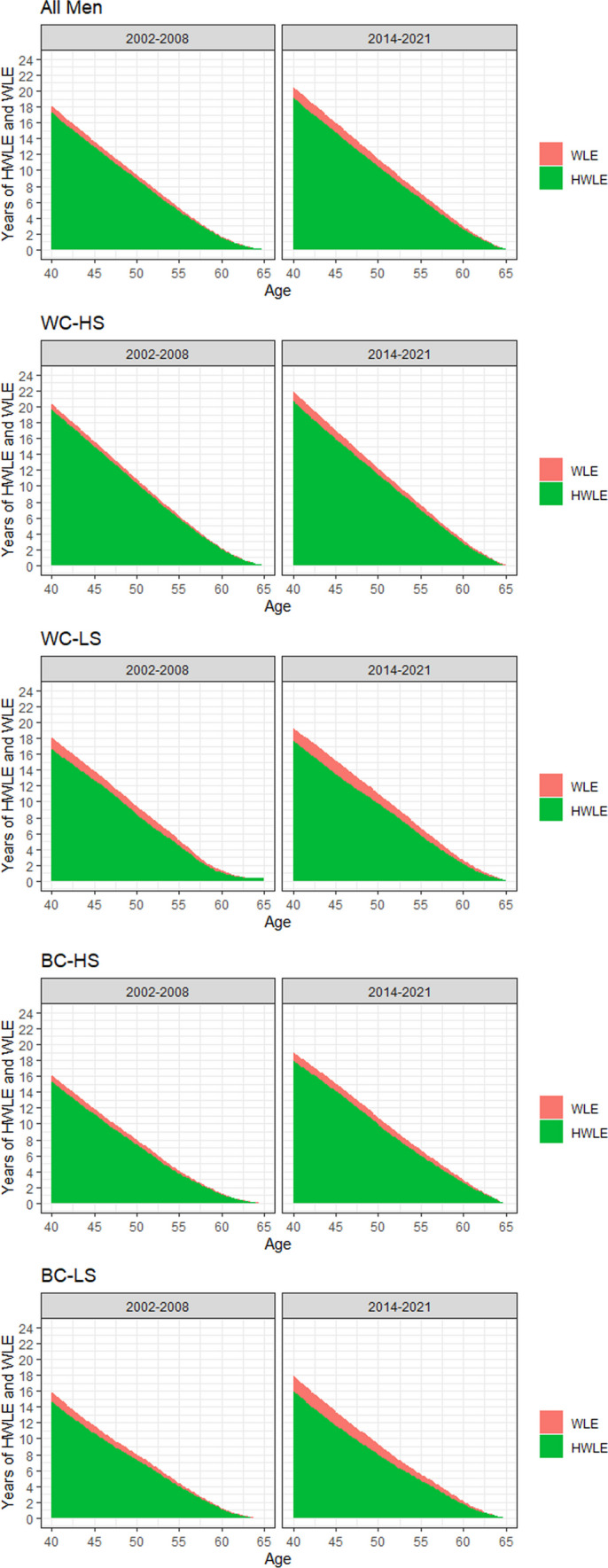
Changes in WLE and HWLE in terms of severe limitations at age 40 and over in men. *Notes*: WC-HS, High skilled white collar workers; WC-LS, Low skilled white collar workers; BC-HS, High skilled blue collar workers; BC-LS, Low skilled blue collar workers

Finally, trends in HWLE and WLE are depicted in Figs. 2 and 3. As can be seen, WLE and HWLE generally increased strongly over time in men and women. At the same time, as also depicted in Figs. 2 and 3, a substantial increase in UHWLE also occurred over time. Proportion of life years participants can be expected to be working without severe limitations at age 40 relative to the overall time spent working decreased only slightly over time from 94 to 91% in women and from 95 to 93% in men. Although trends in WLE, HWLE and UHWLE were similar between occupational groups, absolute values differed strongly. In women aged 40 years, those belonging to the WC-HS occupational group had the highest WLE in the later time period with about 20 years, whereas the WLE in those belonging to the BC-LS occupational group was only about 16 years. In men aged 40 years, those belonging to the WC-HS occupational group had the highest WLE in the later time period with about 22 years, whereas WLE in those belonging to the BC-LS occupational group was only about 18 years. The proportion of life years participants can be expected to be working without severe limitations at age 40 relative to the overall time spent working decreased in all occupational groups: In the case of WC-HS from 94 to 92% in women and from 97 to 94% in men; in the case of WC-LS from 96 to 89% in women and from 95 to 91% in men; in the case of BC-HS from 95 to 87% in women and from 95 to 94% in men; in the case of BC-LS from 87 to 85% in women and from 93 to 89% in men.

## Discussion

We investigated trends in severe limitations among working-age adults using population-based data from Germany spanning the time periods 2002–2021. We found that severe functional limitations remained the same or increased over time depending on the subgroups considered. This increase was most visible in non-working adults in both men and women. Additionally, working life expectancy and healthy working life expectancy were also found to have increased substantially over time. Thus, based on our results, although severe limitations have partly increased in working-age adults, individuals can expect to work more years free from severe limitations. However, the study shows that this development is not due to improvements in health, as no improvements were found in the study population, but that healthy life years are increasingly spent in work. Furthermore, the increase in working life years goes along with an increase in unhealthy working life years over time. This is especially visible in the proportion of life years participants can be expected to be working without severe limitations. In line with the increasing severe limitations, the proportion of life years participants can expect to be working without severe limitations decreased for all, but this decrease seemed especially pronounced in those working low skilled white collar jobs.

These results are in line with previous studies that have reported increasing trends in severe functional limitations and disability among working age adults in Europe and other high-income countries (Beller et al. 2022a; b; Verropoulou and Tsimbos 2017). Our findings are also partly consistent with those of van der Noordt et al. (2019), who analysed trends in general disability and working life expectancy with and without overall functional limitations in workers in the Netherlands. They found that the prevalence of disability in workers increased over time and that workers with disability worked longer. Adding to these findings, we also found increasing levels of severe functional limitations and a general increase in working life expectancy. However, our findings also differ from those of van der Noordt et al. (2019) in some respects. First, we focused on severe functional limitations, which indicate a high degree of difficulty or inability to perform certain activities, while they used GALI which corresponds to a broader definition of functional limitations and includes mild difficulties. Second, we examined differences between occupational groups. In contrast to their finding that similar trends were observed across educational groups we found marked differences in limitation trends across working status and occupational groups. Thus, this is the first study to differentiate severe limitation trends between working and non-working adults and to examine their trends in different occupational groups. Our findings revealed strong differences between these groups, with increases in severe functional limitations mostly occurring in non-working adults. Non-working working-age adults constitute a subpopulation with increasing health impairments.

One possible explanation for these findings is the healthy worker effect (Li and Sung 1999). The healthy worker effect refers to the phenomenon that workers tend to be healthier than non-workers due to a selection process that excludes unhealthy individuals from working. Therefore, non-working adults may have higher levels of severe functional limitations because they are more likely to be limited enough to have stopped working or to never have worked. The healthy worker effect may also account for the increasing trends in severe functional limitations among non-working adults over time, as more individuals with mild or moderate functional limitations may continue working due to economic incentives or social policies that encourage longer working lives, such as is the case in Germany with its pension reforms (Ní Léime et al. 2020). However, individuals with severe limitations might be so limited that, despite incentives and policies, they cannot work. This selection process would leave behind a smaller but more severely limited group of non-working adults.

Another factor of importance to the observed health trends is the changing occupational structure of the working-age population. As shown in Table 2, the proportion of low skilled blue collar workers decreased from 17% in 2002 to 8% in 2021, while the proportion of high skilled white collar workers increased from 38 to 60%. This may reflect shifts in the labour market and changing work demands that could affect the health and work ability of populations (Azevedo et al. 2021; Carr and Namkung 2021; Schaufeli and Taris 2014). The changes in the occupational structure over the past decades may have also influenced the possibilities and constraints for working longer. As the share of low skilled occupations has declined and the share of high skilled occupations has increased, the demand for low skilled workers has also likely decreased and the demand for high skilled workers has increased. This might have increasingly created a labor market, where low skilled workers face higher risks of precarious employment or unemployment, while high skilled workers enjoy more job opportunities (Giesecke et al. 2015). These differences in labor market outcomes might have affected the ability and willingness of workers to extend their working lives: Low skilled workers may have more incentives to work longer to compensate for their lower earnings and pensions, but they may also face more barriers due to their poorer health and lower employability. High skilled workers, on the other hand, may be able to retire earlier due to their higher economic resources, but they may also have more opportunities to remain working due to their better health and higher employability. Therefore, one opportunity for future studies might be to examine the role of occupational factors in explaining the variation in health and the length of working life across subgroups and time periods in greater detail.

### Public health implications

The results of this study have important implications for public health and social policy. Regarding public health, the current study showed that a higher proportion of people work over time, which agrees with previous studies from Germany (Dudel et al. 2021; Heller et al. 2022; Tetzlaff et al. 2022). Increased work participation may have beneficial effects for the economy and for some aspects of people’s health, but the relationship is not straightforward and depends on various factors, such as the quality and conditions of work and the individual’s preferences and circumstances and may even be harmful in some cases (Boissonneault and Rios 2021; Harris et al. 2021; van der Noordt et al. 2014, 2019). Therefore, the increased work participation found in the current study is likely to influence whether people stay healthy longer and a compression of morbidity can occur (Geyer and Eberhard 2022). As such, further research is needed to determine the health effects of the extension of working lives.

Additionally, our results also show increasing levels of severe limitations in working-age adults. This suggests a need for more effective prevention and intervention strategies to reduce the onset and progression of functional limitations among working-age adults, especially among those who are not working (Verbrugge and Jette 1994). Such strategies may include the improvement of health behaviours such as physical activity, healthy nutrition and uptake of screening and rehabilitation procedures (Gehlich et al. 2019; Kuoppala and Lamminpää, 2008; Oakman et al. 2017; Sperlich et al. 2020). However, further effort should be spent on providing more equal access to rehabilitation services, as access is often limited and unequal, depending on the availability, affordability, and quality of the services, as well as on characteristics of the potential users and providers (Götz et al. 2020; Wiklund et al. 2016). From a policy perspective, the results also question the feasibility of further extending working lives without addressing the underlying causes of severe functional limitations among the working-age population (Harris et al. 2021). In our study a high prevalence of severe limitations of 10–20% has been found among middle-aged adults with increasing time trends (Tables 1 and 2). According to role theory, work can provide individuals with identity, meaning, and social integration, which can enhance their health and well-being (Barnett and Gareis 2006). Adults with severe limitations, however, are likely to be significantly hampered in performing their work activities or begin working again. Therefore, stronger work support is needed to better accommodate adults with severe limitations. Additionally, public policies might play a crucial role in promoting healthy employment of people with severe limitations, by providing incentives and support, as well as protection against discrimination (Hanga et al. 2015; Krahn et al. 2015; MacEachen et al. 2017). These policies should aim to create a more inclusive and accessible labour market, where people with severe limitations can contribute to and benefit from work.

### Limitations

The current study has some limitations that should be taken into account when interpreting the results. One of them is the lack of mortality data in our data source, which prevented us from calculating true (healthy) life expectancies; instead, our HWLE analyses are conditional on survival (Boissonneault and Rios 2021; de Wind et al. 2018). Secondly, we did not include institutionalized adults, so the true level of severe limitations is likely to be underestimated. Similarly, systematic sample bias resulting due to differential response rates might systematically bias our results, especially since severe limitations might be especially relevant to survey participation (Beller et al. 2022a, b). This is especially relevant for the last obtained survey wave of 2021 which only included repeat-respondents. Similarly, for the Sullivan HWLE analyses, we needed to combine data from two successive waves to increase the sample size and avoid having insufficient data for some subgroups, such as women working in BC-HS jobs. This may introduce some overlap in the participants, as the 2021 wave consisted of repeat respondents. Thus, the current study might underestimate increases among severe limitations over time. However, in a robustness analysis, similar trends were observed when only baseline first time respondents were used (Appendix Fig. 4), further supporting the main finding of increasing severe limitations in working-age adults.

While the Sullivan method has the advantage of being widely used, it also assumes stationarity, which might not hold in reality. Therefore, the results obtained by the Sullivan method may not completely reflect the health status of the population. A more sophisticated approach for future studies would be to use multistate models (van den Hout et al. 2019). Future studies might also include information on disability pension, which could be another important factor for further differentiating the trends in severe functional limitations among working-age adults into those fully working, partially working, not working and receiving a disability pension. However, complicating this topic for our study, the relationship between disability pension and work status in Germany is not clear-cut, as some individuals may receive disability pension and still work actively a few hours, while others may receive disability pension and not work at all (Vanella et al. 2022). Therefore, we decided to use a dichotomous approach as a first step in operationalizing work status. Further studies should strive to better capture the complexity of work arrangements and health over time.

Finally, we only used self-reported limitations as our outcome variable, which is subject to self-report biases. Future studies could replicate and extend our results by employing other data sources, such as routinely collected data from pension insurance of health insurance providers. Another limitation of our study is that we could not distinguish “never workers” from non-responders in our study. However, this likely does not affect our conclusions, as similar results were obtained in a robustness analysis when excluding those with missing occupational group information (Appendix Fig. 5). One final limitation of our study is that we could not meaningfully compare our estimates of WLE and (U)HWLE with the partial life expectancy for ages 40–65, as obtained from population mortality data. This would have allowed us to assess the extent to which the changes in WLE and (U)HWLE reflect changes in overall survival or changes in work participation and health status. However, in Germany there are no statistics on partial life expectancy based on socioeconomic subgroups, which would be necessary to make a meaningful comparison with our results given the substantial occupational variations in WLE and HWLE. If the partial life expectancy is found to be longer in more recent time periods, this could contribute to increases in WLE, as for example observed in this study. Future research could try to perform such analysis using data from other sources, such as cohort studies or administrative records to complement our approach.

## Figures and Tables

**Fig. 4:**
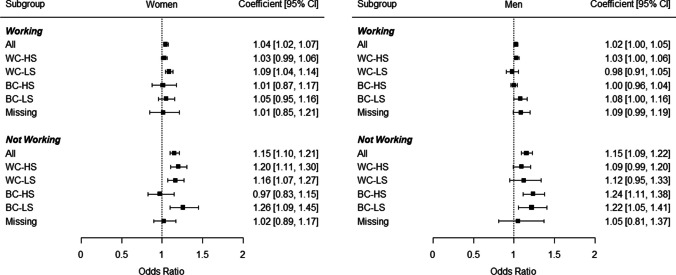
Trend coefficients for severe limitations in Germany for working-age adults across subgroups for first respondents only. *Notes.* Analyses were adjusted for age. WC-HS, High skilled white collar workers; WC-LS, Low skilled white collar workers; BC-HS, High skilled blue collar workers; BC-LS, Low skilled blue collar workers; Missing, Missing occupational information

**Fig. 5:**
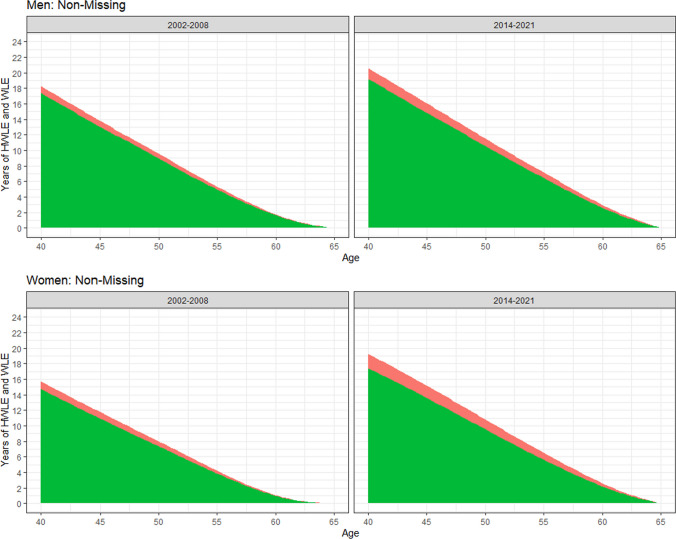
Changes in WLE and HWLE for Women and men with occupational group information. *Notes:* Participants with missing occupational group information were excluded for this analysis. Analyses were adjusted for age

## Data Availability

The data underlying this article are available from the Research Data Centre of the German Centre of Gerontology at https://www.dza.de/en/research/fdz.

## Appendix

See Table 3 and Figs. 4 and 5.

**Table 3:** Severe limitations and socio-demographics in german working-age adults across working status

	Stratified by working status	
	Not working	Working
N	3788	7827
Severe limitations (%)	23.9	7.4
Age (mean (SD))	57.91 (7.00)	52.18 (6.51)
Gender = Women (%)	58.0	49.4
Education (%)		
Lower	12.1	4.8
Intermeditate	58.5	50.0
Higher	29.4	45.2
Occupational group (%)		
WC-HS	38.3	53.8
WC-LS	24.0	19.5
BC-HS	15.5	12.3
BC-LS	16.0	11.6
Missing	6.2	2.7

WC-HS, High skilled white collar workers; WC-LS, Low skilled white collar workers; BC-HS, High skilled blue collar workers; BC-LS, Low skilled blue collar workers; Missing, Missing occupational information
